# Shored curfews: Constructions of pandemic islandness in contemporary Sri Lanka

**DOI:** 10.1007/s40152-022-00262-5

**Published:** 2022-03-10

**Authors:** Vichitra Godamunne, Azhar Jainul Abdeen, Rapti Siriwardane-de Zoysa

**Affiliations:** 1 Colombo, Sri Lanka; 2 Rural Economic and Community Development Organization (RECDO), Kantale, Sri Lanka; 3 Department for Social Sciences, Leibniz Centre for Tropical Marine Research (ZMT), Fahrenheitstrasse 6, 28359 Bremen, Germany

**Keywords:** Island imaginaries, Pandemic governance, Quarantine and curfew, Small-scale fisheries, Coastal Sri Lanka

## Abstract

This paper explores COVID-19 pandemic biopolitics in Sri Lanka through tropes of “islanding” and segregation by discussing how notions of island isolation, insularity, and geo-spatial boundedness have been transformed from their colonial origins to our post-colonial present, and in the wake of wartime governance. We engage with interlocking notions of the “pandemic island” and the “islanding” of a zoonotic virus with which to broaden relational thinking on local pandemic realities. We argue that the pandemic has tacitly shaped imaginaries of oceanic “islandness” in contemporary times by focusing on five interrelated island(ed) tropes in the humanities and interpretive social sciences against the context of the pandemic. These include the carceral (fortressed) island, the utopic island, the “urban” island, the illicit island, and the mythologised (cursed) island. This paper further contributes toward an understanding of contemporary islands and island imaginaries, an understudied dimension of pandemic-related land-sea sociality.

I’ve always believed that when I die, it will be a death out at sea. Yet being grounded

indefinitely during quarantine curfew seems like a slow death. The sea is my life, my very being.

*–* Mohamed Shafeer, gillnet fisherman from Muttur, eastern Sri Lanka (March 2021)

...perhaps without exception [quarantine was] ...the most gigantic, extraordinary, and mischievous superstructure, that has ever been raised by man, upon a purely imaginary foundation.

*–* Dr Charles Maclean, 19^th^ century anti-contagionist advocate (cited in Booker 2016: 12)

## Introduction

What might it mean to be an oceanic “island”—particularly in the majority world—during times of the COVID-19 pandemic? Island(ed) geographies, from post-“Enlightenment” and modernist perspectives, have long stood to represent opposing allegories—of land and sea, as spaces of forced exile and idyllic retreat, of isolation, insularity, and connectivity, and of strangeness and hospitality, among a host of other opposing utopian and dystopian dualisms. Among these, the secluded figure of the “quarantine island” as a form of socio-spatial and temporal containment bears intrinsically maritime meanings of mobility, sojourn, and border traversing (Tuncbilek 2020). For example, island littoral sites were among the first to witness new socio-technical practices such as mass fumigation for the control of potentially contaminated cargo and crewmen (see Engelmann and Lynteris 2020). Such spaces of forced isolation and sanitization were transformed into sites of exception, far removed from ordinary shored life.

The outbreak of the COVID-19 pandemic ushered dynamics that have shaped the biopolitics^1^ of contagion, global healthcare, and the governability of everyday socio-cultural life. Intensely securitized modes of surveillance and governance all over the world have followed in its wake—influencing mobilities, circulations, patterns of production and consumption, the very norms and practices of everyday sociality, and their emotional climates of hope, fear, privilege, and marginality. Meanwhile, newly emergent pandemic realities ruptured ways in which timeworn North-South distinctions were being storied and re-enacted through the making of global metrics, new epicenters of risk, and travel corridors. Fast-moving geographies of flow and contagion translated into real material circumstances, such as unprecedented border closures, travel restrictions, inoculation dogmas, hierarchies, and zones of exclusion that were politically flecked, as much as they were biomedically determined.

One of the more curious discourses to have emerged from popular geo-political imaginaries on contagion was that of islands in relation to that of security and national containment. Island geographies and their metaphoric renderings are unique in two ways. First, they reflect center-periphery relations of (post)colonial power against narratives of smallness, isolation, and dependence (Hau’ofa 2008). Second, the conceptual link of islands with territorial boundedness contributes to “their use as convenient laboratories for studying disease,” yet the relationship between “islandness” (as identity and process) and contagion remain deeply ambivalent (Grydehøj et al., 2020). Island spaces draw on similar epidemic control practices as do their landlocked counterparts, while seeming to possess unique geographical advantages such as closer bonds of social capital and the ability to better police human mobility (Baldacchino 2004). Yet, the intersecting complexities of island geopolitics and COVID-19 biopolitics put to question these geographically deterministic perspectives through markedly diverse trajectories of arrival, contagion, and control (Boyd et al. 2020; Herman 2020).

This paper examines how rapidly transforming pandemic realities over the past year and a half have colored internal imaginaries and enactments of “islandness” through practices of social in/exclusion, belonging, and everyday interaction during pandemic times—rather than how island geopolitics have been shaped by the unfolding of a pandemic. As a first step, we critically consider the curious figure of the “pandemic island” against their associations with quarantine life—a distinct historic and transcultural practice itself. We turn to contemporary Sri Lanka for inspiration, given its complex, multi-stranded histories of geopolitical “islanding,” the revival of colonial epidemic legislation, wartime and post-war surveillance, and the refashioning of curfew from a means of civic control in the past to that of a biopolitical practice by the state. Our analysis is situated against the backdrop of an ongoing national fiscal crisis that offers an additional layer of complexity in understanding the cross-border flows of people, aquatic beings, microbes, goods, capital, and other material flows.^2^

In reading pandemic inflections “through the sea,” we unpick these very antithetical worlds of quarantine as distinct forms of islanding in themselves. Our discussion meanders through divergent littoral worlds—of shoreline quarantine facilities, tourist geographies, urban seafood markets, and illicit fishing livelihoods in shadow economies. Five pervasive tropes are enlivened in this analysis: the carceral island (through quarantine fortressing), the secluded utopic, the un/making of urban islands, islands of illicitness, and of mythologically sacred/cursed island, all of which to varying degrees have dominated metaphoric and historical imaginaries of “islandness” (see Klooster 1998; Mountz 2011; Luo and Grydehøj, 2017). We further ask where such readings of oceanic islandness begin and end during a pandemic, particularly when meanings and practices of emplacement, belonging, dislocation, and (im)mobility continually shape-shift and transform.

### “Pandemic islands”: conceptual and empirical currents

Islands remain one of the most paradoxical nature-cultural concepts to define and theorize. As Hayward (2016: 1) argues, “in one sense, islands are easy to characterize” if taken at face value for their biogeographical form, as bodies of land bounded by water, while never being entirely submerged during high tide. But the presence (or absence) of water itself proves problematic, as natural islands have over millennia been de-islanded and conjoined with land masses through engineered technical processes of bridging, dredging, and land reclamation. From a socio-cultural perspective, the reading of islands as container spaces, or as “densely cultured territory” (Suwa 2007: 7) renders problematic in its multiple associations with smallness, isolation, biological endemicity, and insularity. Yet if islands were to be metaphorically read as cultural landscapes, drawing from the Japanese/*Ryukyuan* concept of *shima*, then, all communal spaces can also imaginatively be read as islands (ibid: 6). Thus, the terms “islandness” and “islanding”^3^ prove useful in exploring the geo-cultural and biopolitical dimensions of reconfiguring island spaces during pandemics, and the discursive construction of pandemic islands themselves.

Curiously enough, the nomenclature of the novel coronavirus pandemic was at times flecked with a number of sea-borne-related metaphors. The most visible of these was the allusion to metaphoric “waves” of arrival and contagion as if they were windswept across shores. Islands too were figured in pandemic imaginaries, as media-fed and scientific narratives on exodus and enclaving evinced singular islands in two paradoxical ways. First, during the early days of the COVID-19 outbreak, particular island states were mooted as getaways “to survive pandemics” (see Osborne 2020). Geographically deterministic narratives on the benefits of sea-locked isolation(ism) were implicit in the early days of the pandemic, when referencing large island spaces such as New Zealand, Iceland, and those in the Caribbean despite their airborne connectivity. On the other hand, the archipelagic expansiveness of countries such as Indonesia and the Philippines discursively rendered their shored edges and landing sites permeable and thus challenging to govern. Arguably then, islands were perceived paradoxically as both secluded and highly infectious owing to their “insular” geographies and varying population densities.

Yet, it could be asked how pandemic realities both enlivened and possibly challenged meanings of “islandness” (see Hayward 2016) across diverse archipelagic spaces such as the Caribbean and maritime Southeast Asia. How was everyday local littoral life transformed in ways that both reinforced and rejected their territorial boundedness and their own antipodal constructions of island(ed) sensibilities and identities? It is also worth revisiting the question of why islanding as a socio-political process matters in the theatrical “geopolitics of disease” (Ingram, 2009). Indeed, through readings across the humanities and the social sciences, the figure of the “island” has offered abundant inspiration to reflect upon and trace manifold littoral worlds—from utopia and paradise, to the captive and extractive spaces of slavery, labor, and resource exploitation.

Yet, islands have been more than mere metaphorical theory-machines, mirroring societies and, their value-laden imaginaries. Islands—in both material and metaphoric senses—have served as microcosms of colonial encounters and sensibilities. Often the figure of the island (whether a speck in an archipelago or a continental land mass) features prominently in imperial imaginaries invoking hackneyed representations of Colombian landings and “native” communities on pristine shorelines. Islands also fell under the same binary-laden reductive gaze, for example, when depicted in Césaire’s (1969) postcolonial Caliban in his critique of the Shakespearean character symbolizing the tribal, the beastly, and the primitive inhabiting *terra nullis*, an imaginatively remote peripheral space that history left behind. It is this notion of “geopolitical belittlement” that Hau’ofa (2008) writes of which not only legitimates and reinforces perceived island imaginaries of smallness, insularity, and isolation. Indeed, the fictitious nature of remoteness and of capsular island dependency has been well revealed throughout histories of empire, land dispossession, and resettlement as they have long been used to establish military bases, reservations, quarantine spaces, post-war nuclear testing sites, and offshore (jurisdictional) zones of exception and internment.

Moreover, as recent multidisciplinary developments such as island studies, together with research on urban archipelagos and “aquapelagos,” have been gaining increasing traction (see Baldacchino 2004; Bremner 2017), the expansive range of often ambivalent and multi-stranded meanings of islands—in all their diversity—are yet to be more comprehensively explored. As John Gillis posits, “islands evoke a greater range of emotions than any other land form representing continuity and separation, paradise and hell, connection and isolation, vulnerability, and freedom being as it were “the West’s favorite location for visions of both the past and future […] origins and extinctions” (2004: 3).

i. *Islanding and island(ed) epidemics*
  From a *longue durée* perspective, the poetics and politics of quarantine and containment have been intimately associated with maritime life and islands in particular. From cargo, ship crews, and indentured labor, to secluded enclaved spaces of internment, quarantine has been used as a means of socio-spatial control, racialization, classed, and gendered stratification. As previously stated, we refer to islanding here as an imaginative and geopolitical process in ways similar to quarantine itself. Space and social relations within islands itself assume a container-like quality, producing different kinds of spatial and relational configurations.
  Thus, philosophically and historically, islands have long stood as a reference point for bridging two antithetical notions of space: the oft-studied utopic and the quarantine-contaminated and the carceral. Both these spaces are exemplarily made and far distanced from quotidian life (Christou and Farmaki 2019). Booker (2016) in his volume on maritime quarantine spanning 400 years of British colonial expansion poses the question as to why most contemporary historians had overlooked the practice of quarantine for its definitive history in shaping society. Since the Black Death-ravaged 14th century, the practice of quarantine had its own discontents as it was a costly affair; condemned cargo and entire ships were set aflame. Etymologically, its modernist meanings bear Venetian origins. *Quaranta* stood for its 40-day duration of sequestration and/or seclusion on vessels anchored out at sea, before they were allowed ashore.
  Indeed, the practice of communal isolation for diseases such as leprosy had been practiced in some form cross-culturally over millennia, long before its pervasiveness in the medieval Christian and Islamic worlds. By the early 1400s, Venice converted an island into a quarantine colony (known as a lazaret), replete with its Biblical reference to the man who was raised from the dead. This namesake peppered much of later historical spaces. Take for example Philadelphia’s Lazaretto Hospital, built outside the boundaries of the city where several thousand Yellow Fever patients perished; or Singapore’s Lazarus Island nestled in a cluster of southern islets that were once one of the largest quarantine facilities of the British Empire in the 19th century where exiles, convicts and indentured labor were interred.^4^ However, in a contemporary context, islands and disease have remained conceptually distant. Apart from their association against the neoliberal economics and geographies of tropical tourism, islands continue to be imaginatively linked to spaces of internment such as historic prisons (e.g., Alcatraz Island) and refugee detention centers, all of which are embedded within a broader geopolitical matrix of border control and policing.
ii. *Denaturalising Sri Lanka’s islandness*
  Our choice of Sri Lanka as a case study may appear counterintuitive. Sri Lanka does not offer to be read as an Indian Ocean “island society” due to its expansive hinterlands, and it would seem erroneous to compare its littoral milieu to other spaces such as the Maldives or the Seychelles. Moreover, apart from its resplendent palm-fringed, lagoon-laced representation in tourist depictions, the sea exists as a shadowy backdrop in Sri Lanka’s popular cultural imagination and folklore. Marine epistemologies and everyday interaction with the sea remain circumscribed to particular littoral groups such as fisher and diving collectives (Siriwardane-de Zoysa et al. 2021).
  While critically considering how its “islandness” is lived and represented, we embed this work within recent critical scholarship that has called to attention the very constructed nature of Ceylon/Sri Lanka as an islanded container space, by tracing its making as an imperial project and postcolonial construct. As Jazeel writes, “like all geopolitical facts [...] the Sri Lankan Island is also a mapping; a way of seeing and imagining space that itself has a representational history” (2009, 400). The seeming naturalization of Ceylon as an island is similarly questioned in Sivasundaram’s (2013) historic volume on colonial British state-making. Drawing attention to how Ceylon was “partitioned and islanded,” not only in terms of the ways in which it was ruled but also in relation to how “native” knowledge of those that were governed came to be co-opted and naturalized as privileged imperial knowledge.^5^
  Like other islands that stood at the crossroads of maritime trade, Ceylon/Sri Lanka had sequential colonial trajectories. Official historic narratives call attention to its strategic location housing a series of pre-colonial trading ports along the maritime Silk Route, as a garrison crown colony governed separately from British India, to a contemporary island-state. Indeed, the sea and its contested coastlines have featured prominently in Sri Lanka’s multi-stranded history. The protracted armed secessionist conflict (1983-2009) transformed coastal and maritime spaces of Sri Lanka into resource frontiers and territories that were to be primarily fought for and fought over. The everyday imaginaries of littoral communities whose lives were intimately bound to the sea have seldom been given much recognition in the island’s meta-histories and geographies.

## Methods and materials

This multi-sited collaboration offers to be taken as a recent kind “connected ethnography” for times of immobility (Sridhar and Jalais 2021), and as a means of wayfaring through our different socio-spatial emplacements and linguistic positionings that are privileged, partial, and at times contradictory. We draw on our auto-ethnographic insights from Sri Lanka’s littoral northeast and its commercial capital Colombo during moments of transcontinental travel and border crossing between March 2020 and September 2021. Our reflections are further supplemented by a select number of in-depth interviews and small informal focus group discussions conducted with fishing communities that unfolded between phases of lockdown and when movement was made permissible in the north-eastern coastal sites of Muttur, Kinnya, and Kantale.

The three authors are Sri Lankan by descent. The first author is an independent writer and communications consultant based in Colombo with a formative background in film studies and literary criticism. The second is a political science-trained NGO co-founder, community educator, and researcher-activist residing in Trincomalee, with a long history of facilitating inclusive community-building projects during wartime and its aftermath. The third of us is an environmental anthropologist and a transnational researcher who spent the first year of the pandemic working remotely between Germany and Sri Lanka. The first and third authors work across English and Sinhala, while the second author works in Tamil and Sinhala.

For a number of reasons, collaboratively co-thinking and writing this paper proved circuitous. In part, this is due to rapid changes on the ground in Sri Lanka over a series of lockdowns. The events captured in this paper offer fleeting snapshots in time against trajectories of pandemic governability and the politicization of COVID-19. Neither does our writing claim to represent a particular social collective or geographic space. We use our own embodied sensibilities in reflecting on loss and grief on the one hand, and the confrontation of our own positions of privilege, (un)certainty, and epistemic (dis)advantage as researchers far removed from our own “field sites” while struggling to make sense of and responding to diverse forms of precarity (see Grewal 2021).

This early sense of site-bound (un)knowability proved uncanny in several ways. Never had such a period of state mandated shut-in been sustained under curfew orders for so long, while sensibilities akin to quarantining-in-place is arguably nothing novel to the lives of many Sri Lankans who have come to naturalize the enforcement of curfew over decades of civil war. Yet, it was this particular kind of containment that bore meanings of ambivalence, for curfew was often a collectively anticipated political fact/event during or in the immediate aftermaths of civil-state violence. In the former war-ravaged northern and eastern areas controlled by the state military, curfew was experienced in the form of enduring time bound restrictions on mobility after dark. These familiarities and dissonances will be further explored in the context of ordinary littoral life, particularly among fisher communities in the northeast.

The shape-shifting nature of this study also owed much to our own cross-examinations with respect to inherent biases and blind-spots, given our highly situated and partial experiences, for what was left un-witnessed and by whom, remain equally salient. Questions that patterned our own meaning-making were often rooted in making sense of pandemic governance, emergency provisions, and autocratization processes which were felt in ways that were disproportionate to the severity of particular localized contexts, and the nuances inherent in “factual” realities that were in daily flux. In such instances, one’s own body becomes a multisensory site of “field” praxis and engagement (Siriwardane-de Zoysa et al. 2021).

Yet from an epistemological perspective, what appeared deceptively “universal” however was the virtual representation of pandemic life that was controlled and mediated by private and state-owned media newsrooms, as officialized authorities whittled to few limited voices comprising those of military, police, and Health Ministry spokespersons. How redundant was print media as discursive source material, at a time when state rules on lockdown, their ease or prolongation were sporadically announced the night before? These pointed toward the transience of our corpus, as newsflashes were constantly re-tinkered *ex-ante.*

With the limited means of data collection, we rely on qualitative analysis combining: (a) unsystematically sampled local news articles in print and digital media across English, Tamil, and Sinhala between January 2020 and September 2021; (b) two focus group discussions^6^ among 15 small-scale coastal fishers (gillnet and long-liners) together with ornamental fish divers in Muttur and Kinniya respectively (11 men and 4 women); and (c) three in-depth interviews with female dry-fish makers and sellers. The participants comprised members of fisher cooperatives that the second and third author had formerly partnered on other projects.

## “Islanding” a pandemic: poetics and practices of antithetical quarantine worlds

In contexts of viral invasion and contagion, their stories of arrival—whether across land or sea—bear significant material and symbolic resonance. High-sea vessels were “among the first identified sources of global contagion” given tight living and working conditions of crew members and passengers (Havice et al. 2020: 655). Moreover, the sea and the politics of microbial containment have been intimately bound, as witnessed in early struggles over voyaging cruise-liners and other shipping vessels that were refused safe harbor and crew member changes during border closures. In reflecting on the plight of seafarers, if being “locked in” was intrinsic to sea life, what made the “experience of the pandemic so challenging at sea is being ‘locked out’ of land?” (De Beukelaer 2021). What else does the sea and the politics of containment convey with regard to other kinds of human, more-than-human, biopolitical, and technological entanglements? Islands, island imaginaries, and enactments have been a relatively understudied dimension of pandemic-related land-sea sociality.

Over the course of a five-hundred-year imperial trajectory, Ceylon/Sri Lanka bore witness to multiple epidemics, given in part to its placement along east-west maritime trade routes. These included frequent outbreaks of diseases such as malaria, yaws, smallpox, cholera, and tuberculosis (Meegama 2012). While colonial medicine shared a complex entangled history with the island’s indigenous medicinal knowledge and practice (Sivasundaram 2013), the systemic modernization of healthcare unfolded in the latter half of the 19th century^7^ in the wake of Universal Adult Suffrage in 1931. Following independence from British rule, a fully state-funded universal public healthcare system was established in 1977 with the abolition of user fees and financed by public taxation,^8^ strengthened by early liberal democratic rule.

Tellingly, when the novel coronavirus was first declared by the WHO in January 2020, there was much concern on overburdening a resource-strapped public health system. Until the first quarter of 2021, Sri Lanka had relatively fewer COVID-19 infections. The treatment of COVID-19 cases was restricted state-run hospitals and government medical staff at the start. This was made possible by the revival of imperial epidemic legislation legally mandating that all infections had to receive state medical attention, while requiring the institutional isolation of anyone infected or suspected to be carrying the disease thus making a new kind of biopolitical subject,^9^ a practice that was later abandoned after the exponential increase of new infections toward mid-2021.

i. *Carceral becomings: enactments of the island fortress*
  The porosity of Sri Lanka’s borders became a discursive battlefield primarily because national infection metrics mattered and each landed flight foretold the likelihood of increasing headcounts in infections. Centrally-controlled state messaging that was amplified by the local media apparatus used vocabulary which emphasized pandemic nationalism and paternalism. The securitized narratives were reminiscent of wartime discourses of fear, uncertainty, and shared enmity.^10^ Sensationalist media reports fanned a climate of fear by public shaming of individual patients and racialized accusations of entire communities^11^ for not adhering to health protocols and endangering an entire nation. The metaphor of the “invisible invader” was initially associated tales of contagion from recalcitrant working class Sri Lankan foreign returnees and municipal neighborhoods that were often disproportionately policed, criminalized, and subject to draconian forms of public punishment and castigation (see Pieris 2021b).
  The framing of the COVID-19 pandemic as a crisis of securitization as opposed to a mere health emergency foretold of how deeply entrenched military actors^12^ would become spearheading trajectories of planning, response, and preparedness to varying degrees around the world.^13^ The “new COVID-19 related military-civilian assemblages” became pervasive, from national militaries taking on wide-ranging roles from border control, setting up of field hospitals, to the dispensing of health services and lockdown enforcement at large (Gibson-Fall 2021). Much of Sri Lanka’s post-independence history however has been colored by an Agambenian “state of exception” than that of peace^14^ through lengthy trajectories of anti-insurgent and counter-terrorism laws and restrictions (Pieris 2021a, 2021b). Therefore, what was witnessed in the wake of COVID-19 was a parallel structure of the medica-militia which legitimated a distinct form of pandemic governmentality that had barely “triggered a state of exception anew, but instead has extended the state of exception that the country’s democracy has been functioning in for the past several decades” (Pieris 2021a, 2021b: 20), through well-accustomed cycles of surveillance, arrest, and prolonged detention.
  What was arguably different, however, was the fact that Sri Lanka was being governed in ways that were akin to a historic quarantine island. A two-week quarantine phase was often weaponized as a temporary prison sentence for curfew violators. The technologisation of its security apparatus favored ocular-centric practices, replete with the show of heat scanners, rapid antigen testing “islands” along highways to deter long-distance travel, and an economy of digitized certificates for those who had completed institutional quarantine. Yet, their application was summarily dismissed for political and economic elites and was often sporadically implemented. Thus, an “island” reading of the workings of quarantine fortressing and the making of carceral spaces prove illuminating in several ways.
  First, they invoke the production of a particular kind of dis-*eased* body and island(ed) subject-to-be governed in a liminal space such as quarantine encampment.^15^ Resorts and quarantine facilities that were state-run were equally fortified—with visible armed security patrolling their grounds.
  Second, they also produced hauntologies^16^ of wartime violence in the recent past. For example, the institutional resurrection of particular corporeal practices at border checkpoints replete with identity cards in show of legitimate mobility during lockdown bore echoes of wartime municipal governance. A fellow passenger drew parallels between medical ambulances and notorious political “white van” abductions (see Wickramasinghe 2010). Another referenced the routine rounds military personnel made each day for temperature checks, alluding to the fact that not too long ago they would have held “a very different kind of gun” to the heads of civilians.
  Read together, Sri Lanka’s diverse and often coastally emplaced worlds-in-quarantine remained enmeshed in questions around citizenship, social exclusion, and the reproduction of class, ethno-racial and regional hierarchies. The next section explores the interplay of the island-utopic in the thwarted geographic imaginaries of pandemic tourism.
ii. *Whither the tropical utopic: the (un)making of cultural outsiders*
  Arguably, more than any other recent event, the pandemic foregrounded Sri Lanka’s ambiguity toward its cultural outsiders, particularly as a tourism-dependent economy. At first glance, Sri Lanka’s islanding dynamics were rendered visible through its protracted closure of national borders often to its own citizenry, as thousands of migrant workers particularly in the Middle East had no access to repatriation flights and received little support from local consulates. The first cases reportedly implicating community spread had its origins in the tourism sector, having been traced back to an Italian tour group in March 2020. At the time, everyday racializations of the virus transformed as abruptly as global epicenters did, and the last tourist arrivals became increasingly othered in reference to the “dirty west.” Almost a year later, so-called “bio-secure travel bubbles” were sporadically implemented for VIPs, politicians, and those within political networks, while designs were underway for formulating a new visa category in facilitating remote-work stays and for attracting digital nomads from the global north. In tandem, health authorities beseeched locals to not interact with foreign tourists, to the extent possible. Those that did, including tour operators, drivers, and hotel staff were indiscriminately made to self-isolate themselves for a fortnight. How then can island imaginaries within this dualistic frame of the paradisal and of life-in-quarantine be reframed?
  Island geographies and cultures, as Murray (2009) posits, privilege the “idealized, insular and vulnerable” that legitimates both conquest and its enduring management and control. The question of cultural islands and how they come to be reproduced, normalized, and challenged remain a core construct of “islandness.” The (un)remaking of identities unfolded within insular framings of communal purism, akin to the taxonomic endemicity of biodiversity species, popularized by 19^th^ century-ecological paradigms centered on island biogeographies.^17^ Thus, in the long history of epidemics, the invasiveness of pathogens and human bodies that are perceived as being acquiescent hosts in their circulation, played out through intersectional classed and gendered racializations of disease and the medicalization of race (see Sikka, 2020). Within this curious matrix of (post)colonial islands, the tropicality of the new utopic exodus in the form of neoliberal touristscapes emerges as a distinct figure of the post-COVID era.^18^
  Modern tropical beachscapes in the global south, as distinct neoliberal capitalistic constructs of leisured lifeworlds and excess consumption also serve as exemplary sites. That is to say, they are performatively exemplary despite their refashioned uniformity in the staging of global tourism. The act of being/becoming a “foreign” tourist comes with its own corporeal and affective comportment of outsider-liness, that is often welcomed in sites that service them.^19^ Yet, in pandemic times, formerly “Chinese” and latterly the white body was read as a carrier of contagion, with sporadic incidents of verbal assault, alongside a clutch of tourists who were denied entry into towns by local protestors. Such moments of historic inversion however are fleeting and continue to reproduce and entrench meanings around both exclusive and mass tourism often wedged between two antithetical poles: of sublime isolation and (adventurist) cultural connection, and of geographic remoteness and fixity. Unsurprisingly, consumer experiences that were first frozen during the early onset of the pandemic (see Sheller 2020) are now being capitalized in the form of new travel sensibilities by virtue of enduring global border closures in other places.
  In sum, this chapter engages with divergent worlds-in-quarantine through “islanding” practices containment and the making of its utopic “other,” most visibly seen in the affective geographies of neoliberal tropical touristcapes. These spaces are often littoral, signifying margins and edges of social life. They are of relevance primarily because of their translocal meanings of isolation, seclusion, and proclivity at being fortressed, in creating islands within islands.

## Pandemic islandness: untangling coastal lifeworlds in flux and precarity

For many parts of the world, there has never been a time in history in which the biopolitics of quarantine and curfew have converged in intimate ways. Scholarly work that focuses exclusively on socio-cultural meanings of political curfew and its practices in wartime and contemporary Sri Lanka has remained scant.^20^ In the life of Sri Lanka’s pandemic, meanings of “curfew” are further complicated through what Bianchetti et al. (2020) term as “quarantine urbanism(s).” In Colombo and across much of the island’s urban centers, containment remained paradoxically site-focused despite previous trajectories of circulation among people, goods, and other objects. Regulations were premised on micro-circulations and interactions, whereby sites that were cordoned off included apparel factories, low-income apartment blocks, and at times entire urban quarters associated with higher levels of poverty where mass testing was more rampant. Often, blue-collar workers were institutionally quarantined en-masse with little forewarning of where they were being taken or for how long. Places “condemned” as being contaminated were deemed out-of-bounds, as eventful sites, festooned by quarantine notices put up by public health officials and police.

i. *The “urban island” effect: of fish, markets, and leperised bodies*

Island bio-geographies can be thought of as “urban islands” with which to explore the discursive practices of municipal quarantine. Cities themselves exist as terrestrial archipelagos through their socio-spatial histories of quartering and splintering as “exclusionary enclaves, gated communities, immigrant detention centers, isolated villages and pariah states,” as much as rogue municipalities (ICUA 2015). Their distinct urbanities are embedded in a quarantine matrix of flow and fixity, core, and periphery. The socio-morphological re-ordering of city spaces and circulations can be witnessed not only in restricted mobility but in the socio-spatial containerisation of everyday life.^21^ For example, in demonstrating the state-led success of quarantine control, the discourse of “clustering” was adopted by national media erroneously alluding to the fact that viral circulations were micro-sited, implicitly bound within the walls of particular places after which the clusters themselves were named.

One of the most politicized sites was the Peliyagoda Central Fish Market, a large urban wholesale seafood complexes located in the outer suburbs of Colombo. The tenuous links between seafood supply, trade, and its itinerant workers bear similarities to wet market spaces elsewhere, such as Thailand’s Samut Sakhon Province (see Tan and Lim 2021). To Colombo authorities, the market seemed an expedient scapegoat—crossing trajectories of blame from the terrestrial to the marine. The very fact that Sri Lanka’s medical authorities named the second wave of infections as the “Peliyagoda Fish Market Cluster” sealed its enduring stigmatization.

Moreover, if the bodies of itinerant fishmongers proved amenable to pollutive imaginaries, so did the moist, decaying bodies of fresh fly-ridden catch that was iced and slabbed to be sold. What these corporeal anxieties ushered was a considerable increase in the production of dry-fish over those months.^22^ Subsequently, the othering and mass avoidance of wholesale fish markets rose to such levels of absurdity, implicating a former Fisheries Minister who televised himself biting into a raw fish during a press conference in a desperate bid to quell fears.

The fact that fishmongers came into contact with bodies that were mobile and itinerant sat well with urban rumors and fears, in which affluent neighborhoods found themselves discouraging vans and lorries selling fresh seafood from traversing their spaces. What was therefore witnessed was the leperization of an entire supply chain and all who eked a living through the fisheries and seafood industry. These reverberations further marginalized small-scale and commercial fish production that had already been subject to market disruptions and a host of other socio-economic dependencies. The next subsection will explore these understudied dynamics that bring to the fore plural coping and adaptive strategies of survival, some of which were contingent on curfew life and others transgressive.

ii. *Of island illicitness and fractured coastal livelihoods*

Stark disruptions and wide-ranging changes witnessed in global and local value chains of seafood supply, production, and market relations were felt in Sri Lanka in similar ways (see Bennett et al. 2020). While there is rich literature on the increase of under-reported and unregulated fishing worldwide, what is increasingly emerging are trajectories of multiple livelihood “dislocation,” deprivation and knock-on-effects stemming from mounting fuel costs, inaccessibly to formal and informal social capital, administrative-bureaucratic slow down.

To many fisher communities across Trincomalee, in Sri Lanka’s war and tsunami-affected northeast, quarantine-curfew came as a veritable surprise. First, because it warranted a leap of faith in dissociating from more recent collective memories of political curfew as COVID-19 was initially barely experienced as a health crisis. Second, because the extension of terrestrial curfew outwards “to the sea” made little sense as boats operated at considerable distances from the shoreline and one another, unless during moments of resource sharing such as food and fuel while afloat. Weeks after the first nationwide lockdown was imposed, a system of fishing permits were implemented through the national Fisheries Department stipulating that each fisherman could access the sea once a week on a rotational basis. These permits were issued through “village”-based grassroots Fisheries Cooperative Societies (FCS), hence, individual membership was paramount at securing such a resource. Landing sites were monitored by the Ministry of Health personnel in tandem with naval forces.

The rotational system of quarantine fishing licensing brought upon diverse implications. For months, fishing and gleaner households including ornamental fish diving collectives found themselves locked into a classic subsistence economy. Among focus group participants, many that fished with gillnets and longlines in coastal waters stated that they had switched to artisanal forms of fishing in lagoon spaces using non-mechanized crafts. These fishing trips were deemed illicit and often took place at nightfall.^23^ The gendered division of labor and production that is prevalent within coastal fisher communities was further characterized by new forms of precarity, as women found themselves dislocated from regular sources of income as nocturnal fishing moved into more perceivably illicit domains. As Sara Umma, a 52-year-old widow from Muttur shared:

I used to wait by the shore for boats to arrive. I could make something by washing their floorboards, collecting fish and cleaning equipment and earned my wage (*kuli*) as a share of the day’s catch After COVID, this became impossible because much of the fishing happened at night. I later obtained some microcredit to start a door-to-door spice business earning between 400-600 LKR.

Mobility and seasonal migration proved near impossible due to district border closures. Livelihoods of illicitness were framed as one of the only means to get by, such as sand mining off the banks of the Mahaweli river that were hauled into tractors (again at night) in loading areas. These occurred around several sites in Muttur, as a former diver in his late 40s and a father of five concurred:

We were told how to take sand from the river… We know no permission was given. I used to earn about 2,000-4,000 LKR on a single night, but you make this kind of money because this is very risky. If you are caught out by the police, you can be individually fined between 10,000-20,000 LKR.

On the other hand, small-scale acts of smuggling on coastal waters (e.g., turmeric, which was in short supply due to import restrictions and narcotics), often implicating smaller island bases and rock outcrops, were described by fisher residents as being prevalent before pandemic times. For some, these supplementary sources of income dwindled as there were fewer boats out at sea with higher numbers of naval patrol vessels. Taken together, sea-bound quarantine curfews determined new contours of “illicitness” amid shadow worlds—closing off some avenues while remaking others.

iii. *Mythologising the pandemic island: between the sacred and the cursed*

In a post-truth world of pandemic (un)making, how are mythological histories and contemporary ever-evolving urban legends invoked to legitimize a particular form of biopolitics? Arguably in part, pandemic governmentality was enabled through an economy of rumors and officialized narratives.^24^ In tandem, a lively trajectory of astrological predictions by soothsayers patronized by politicians including a gamut of Theravada Buddhist faith-based rituals were consistently performed by local politicians in “curing” the island. A curious incident involved the ex-Health Minister who participated in a ritual where she threw a pot of “blessed” water in a river as a symbolic act to heal the nation from COVID-19. The act was heavily ridiculed by the political opposition including the media stating that it was as if she had offered herself up as a sacrifice to absolve an island cursed. This comment was a cursory nod at the legendary queen, Vihara Maha Devi,^25^ the mother (and military advisor) to King Dutugemunu, valourised for reunifying the island under a Sinhalese banner among the Sinhalese community.

Indubitably, the specter of Vihara Maha Devi has been continually invoked at multiple points in Sri Lankan history, while standing to embody Sinhala-Buddhist feminine virtue, ethno-nationalist martyrdom, and the self-sacrificial moral mother figure (De Alwis 2004). Sea sacrifices—often calling upon the feminine virginal—stands as a prevalent trope across a number of transcultural historic legends and lore. Yet, its hauntings in pandemic life reveal meanings of the sea embodying its own recalcitrant sentience, a redeemer of a cursed island, juxtaposed against sacred, divinatory riverine waters that are poured forth as an act of cleansing. Diverse waters have also been implicated in the grievances and injustices around forced cremations, marginalizing Sri Lanka’s Christian and Muslim communities. The ruling party, backed by the Ministry of Health, implied that buried corpses that had succumbed to COVID-19 would invariably contaminate local groundwater (see Abdul Razak and Saleem 2021), thereby constructing another kind of ethnicized pollutive body in its intertwined discourses of viral and moral contamination, shrouded in narratives of un-patriotism. Subsequently, several larger plots of private and state land (of 8-10 acres) in the east coast were designated as burial grounds for communities across the country. Intriguingly, the first spaces to be considered as self-contained burial sites were smaller splintered coastal islands and rocky outcrops off the eastern seaboard, which were abandoned due to their geological infeasibility. These discursive framings once again bear echoes of the carceral island, and in this instance for the interment of the deceased as opposed to the diseased living, symbolizing material sites of secluded decay and racialized othering.

## Concluding remarks

This paper explores a host of diverse island(ed) lifeworlds replete with state-run quarantine facilities, localized tourist bubbles, classed urban neighborhoods, and the disenfranchised rural littoral. In bringing these divergent realities together, we revisit a question posed at the beginning of this paper: where does “islandness” begin and end in the biopolitics of a viral pandemic? Articulations of “islandness” in the public and political imaginary remain relatively untenable at first glance, in which the sea appears as a shadowy backdrop to the unfurling events of the COVID-19 pandemic in Sri Lanka. Yet, when parsing these meanings, two converging themes appear. The first is that of “pandemic islands” in which fears and anxieties over invasiveness, contagion, and bio-cultural endemicity meld in ways that often reproduce meanings of the insular, isolated island. Curfew-quarantine was enlivened as a practice of fortressed control while discourses around clustering particularly in municipal spaces took the form of impermeable land-locked islands themselves.

Yet, constructions of “islandness” also emerge through less visible processes of islanding through which socio-spatial and temporal practices of quarantine and curfew meld. The pandemic has shown that revived historic practices of quarantine are far from being projects at placemaking. Neither are quarantine spaces merely sites of exception, or heterotopias in a Foucauldian sense. Instead, they intertwine with routines of normalcy and everyday life, sustained through multiple interdependencies. What complicates the quotidian are the traces and hauntings of wartime governmentality and their (dis)continuities that are rife in collective memory, entangling diverse sensibilities of inclusion and exclusion, sensory and corporeal meanings of pollution, purity, squalor, and their associative bodies. While contaminative spaces are produced through intersections of class, ethno-racialization, and occupation. Indelibly, these strengthen contours of legitimate control and illicitness in the post-pandemic shadow economy borne as a result of stricter border closures and lengthened periods of site-bound curfew.

In conclusion, this study offers to be taken as an early foray into the poetics and politics of viral contagion and the (un)making of diverse forms of “islandness,” while tracing historic continuities and dissonances, how might contemporary practices of quarantine encampment be put in dialog with colonial spaces of labor internment and contemporary refugee islands, for example, connecting spaces as disparate as Singapore’s Lazarus Island and Otago’s Kamau Taurua? How might different enactments of islandness offer transgressive moments for thinking beyond seclusion and isolation, connectivity, and localization? Do the geo-politics, socialities, and symbolic meanings of islands even conceptually appear relevant in a post-COVID world in which much of what is being governed (and how) is through the making of socio-spatial islands? These tangled themes are by no means exhaustive and offer but a glimpse into the disparate biopolitical lifeworlds of quarantine that oceanic islands with their shoreline edges and metaphoric meanings stand to offer. Taken together, they signify an intricate underbelly, at times revealing (or concealing) what Dr. Charles Maclean, that vocal 19th century advocate of anti-contagionist thought (Kelly 2008), referred to in our opening quotation as an “extraordinary and mischievous superstructure,” congealing particular practices that will endure well after pandemic life.

## Footnotes

1 Biopolitics is taken to mean the politicization, regulation, and economic exploitation of biological bodies. Michel Foucault (1998: 139) introduced the concept of “biopower” to describe the ways in which human beings exist in society as biological entities that are useful to political power According to Foucault, two forms of biopower exist—one that disciplines, optimizes and extorts the human body, and another that seeks to manage populations and eliminate individuals and factors that hinder population management. Giorgio Agamben expanded the discussion of biopolitics by introducing the concept of “bare life” and the division of human biological life into that which is worthy of preservation and that which requires elimination (Agamben, 1998: 77–84). “Bare life” can be conceived as both a political status and as an object of political decisions (ibid).

2 The novel coronavirus pandemic also came in the wake of several political and environmental catastrophes in Sri Lanka – a constitutional crisis (October 2018), the Easter-day bombings implicating a group of extremist youth (April 2019), and the combustion of the X-Press Pearl cargo vessel transporting toxic chemicals off its western seaboard (May 2021).

3 Conventionally, islandness has been interpreted in terms of its bounded spatialisations, and as “a metaphysical sensation that derives from the heightened experience that accompanies physical isolation” (Conkling 2007: 191). Yet, more contemporary, nascent theorisations of the “island hub” complicates the remote and virginal meanings of island spaces as the civilizational Other of larger continent-based landmasses and their historiographies. Readings of island peripherality barely hold much resonance in contexts such as Mauritius, Ceylon, or Singapore that were geo-politically and administratively rendered as islands (or *islanded*), as a result of colonial expansionism.

4 Among the most haunting of these historic sites in recent history is North Brother Island in New York City, now a bird sanctuary with its decaying remnants of the Riverside Hospital in which the infamous "Typhoid Mary" (Mary Mallon) finally succumbed to the disease and died in 1938 (see Payne 2014).

5 If knowledge was a means by which to govern, the dizzying “cosmopolitanism” of Ceylon, as evidenced by one of the earliest British travel writers Robert Percival (Sivasundaram 2013, 21), stands in stark contrast to the racialized identities by which its islanders—many of whom have had long-standing biographical histories of regional sojourn and seaborne mobility— were counted, classified, rendered sedentary, and ethnicised into a handful of ethno-linguistic labels.

6 All interviews were anonymised, and the sample included 6 members of the Muslim community, 4 were Sinhala-speaking fishers (of which two were middlemen) and 5 members of Tamil ethnicity comprising mixed Hindu and Christian faiths. The focus groups were held in both vernaculars – Tamil and Sinhala with simultaneous translation.

7 Ceylon had been transformed into a capitalist plantation economy, witnessing great many circulations—of people, animals, raw materials, commodities, along with new habits, lifestyles, and aspirations. Epidemics thrived as urban areas grew, replete with overcrowded housing and unsanitary living conditions (Meegama 2012: 3–21). The nineteenth century witnessed the movement of people from the Maritime Provinces (previously under the control of the Portuguese and Dutch authorities) to the former previously autonomous Kandyan Kingdom, and the immigration of indentured labor from southern India to work the newly established coffee and tea plantations.

8 Private hospitals grew alongside public healthcare provision. In a similar vein to a number of postcolonial islands and larger coastal states, epidemiology remained a subject of enduring concern, together with the eradication of several infectious diseases such as rubella, polio, measles, and malaria.

9 At the time of writing this paper, over 54 quarantine centers with 10,430 beds, and 92 intermediate centers—for those who had come into contact with COVID patients—with 22,240 beds alone were military-run (Peiris, 2021b: 20). These figures discount scores of resorts and hotel establishments that offered private paid services.

10 The necessity of quelling the first wave was likened to an act of national service, a familiar trope in biopolitical war rhetoric familiarly deployed by other administrations including those of Donald Trump and Boris Johnson.

11 The forced cremation of those who had succumbed to COVID was made mandatory by the state on account that burials would contaminate groundwater, despite WHO guidelines. While cremation has been a practice among Buddhist and Hindu communities, Christians, Muslims, and Jews have traditionally sought burial. Moreover, the act of cremation is doctrinally forbidden in Islam and this policy remained yet another instance in a long trajectory of ‘ethnic minority’ discriminatory practices and marginalization, also in relation to the violent surge of Islamophobia among Sinhala-Buddhist polities—also lead by Buddhist clergymen—in the years following war’s end (Abdul Razak and Saleem 2021).

12 The pandemic unfurled in the immediate aftermath of Gotabaya Rajapakse’s heated presidential election, a former military general and a scion of the Rajapakse family under whose authoritarian rule Sri Lanka’s civil war was brought to brutal military end in 2009, and often stood to represent majoritarian Sinhala-Buddhist ethnocracy (DeVotta 2021). His campaign ticket, thus returning his family into a formal position of power, was that of ensuring “national security” while articulating a strongman image privileging “meritocracy, expertise and efficiency” (Peiris, 2021a: 17).

13 Colonial entanglements between modern medicine, warfare, population control, and interment have been richly documented, and these continuities prevail well into contemporary life, as seen for example cooperative practices and alliances among militaries, international healthcare, and research institutes (Beaumier al., 2013; Howell 2014).

14 Post-war “development” in the north and east remains quintessentially military-led with the placement of retired military personnel in civil service positions since 2009 (Kadirgama, 2013).

15 The consumption of alcohol and cigarettes was officially not permitted in any quarantine facility—whether hotel, private or state-run. Yet, in being transferred to hotel-run facility, one of the co-authors related how after a group briefing with the passengers of her flight in the dead of night, several hazmat-suited military personnel approached a male neighbor occupying the next room requesting whether he had a bottle of imported alcohol that he could gift them or share. Such “gifts” would enable some degree of preferential treatment, mimicking relations of power among the incarcerated and their guards in islanded encampments.

16 We borrow this notion from nascent writings in cultural studies, memory-work, and film studies (see Fisher 2012; Lincoln and Lincoln 2015) to refer to not just “ghosts of the past” but to also to new kinds of spectral emergent spectral becomings (i.e., of places, identities, and practices) that bear strong articulations and traces to lived history, but is also perceivably recast in different ways. It is therefore the discursive framings of these apparent dis/continuities that particularly interest us.

17 This parochial form of unique nativism often translates itself into the modernist, statist construct of ethnicity, for as Eriksen (1993: 143) writes, “cultural islands have barely existed in time, making the prevalence of ethnic boundaries one of its most striking features of insular imagination.”.

18 Consider South Africa’s post-Apartheid dynamics to the re-colonization of beaches following Hurricane Katharina as waterfront properties were progressively bought over displacing older southern black communities that were historically redlined (see Kahrl 2016).

19 The geographies of post-war beach tourism in littoral Trincomalee and Batticaloa are far removed—both spatially and imaginatively—from the infrastructural remnants of wartime, some which have been memoralised by the military for local visitation while indubitably appealing to particular Sinhala-Buddhist polities.

20 The imposition of curfew has always remained the first port of call during political upheaval also in recent years, taking for example the aftermaths of ethno-Sinhalese mob attacks on mosques and the Easter day bombings. In effect, political curfews lasting a few days at a time are often discursively framed by the state as a preventative measure against society escalating toward lawlessness, while in effect, it alienated groups that were most at-risk from their own informal sources of community support, and particularly at times when they were most needed.

21 For example, during nation-wide lockdowns many small-scale grocery stores in urban municipalities remained closed. In moments in which restrictions were informally eased, they remained “partially closed” by dealing with customers in ways that were subversive yet elusive enough to be retracted when called out—“we now know the value of having a backdoor” a grocer in Kantale, once quipped.

22 Large quantities of fish that are presently unknown were salted to be used for the dry fish trade, including species not traditionally utilized.

23 Others, particularly women who were not formerly associated with their family fisher livelihoods, increasingly turned to dry fish making and marketing, a sector with a higher percentage of female participation. Men on the other hand opted for day-wage labor assisting with carpentry and masonry work were cited as other means of supplementing income on 6 days of the week coastal fishing was not permitted. As fishermen, construction sites would often pay half or two-thirds of the value of a regular workman’s salary, amounting to earnings of 700–800 LKR (3.50–4.00 USD) a day.

24 Communiques penned by local medical associations to the Ministry of Health and the general public were rife calling for the unlearning and debunking of ‘myths’ regarding the spread and curative potentialities of COVID-19.

25 According to the Theravada Buddhist chronicle the *Mahavamsa*, Vihara Maha Devi as a young princess offered herself as a human sacrifice to the ocean to placate the angry gods, after her father, the king, slew a young Buddhist monk who was wrongfully accused of a sexual liaison with his consort. Unlike the Greco-Roman Andromeda who was demanded as an offering for the folly of a conceited mother, the princess acting out of her own volition was celestially pitied and spared by the gods themselves.

## References

[CR1] Abdul Razak, Mohamed Imtiyaz & Saleem, Amjad Mohamed. 2021. ‘COVID-19: the crossroads for Sinhala–Muslim relations in Sri Lanka.’ *Journal of Asian and African Studies*, 00219096211021873.

[CR2] Agamben, Giorgio. 1998. Homo Sacer: Sovereign Power and Bare Life. Translated by Daniel Heller-Roazen. Stanford, California: Stanford University Press.

[CR3] Baldacchino Godfrey (2004). The Coming of Age of Island Studies. Tijdschrift Voor Economische En Sociale Geografie.

[CR4] Beaumier Coreen M (2013). United States military tropical medicine: Extraordinary legacy, uncertain future. PLOS Neglected Tropical Diseases.

[CR5] Bennett Nathan J, Finkbeiner Elena M, Ban Natalie C, Belhabib Dyhia, Jupiter Stacy D, Kittinger John N, Mangubhai Sangeeta, Scholtens Joeri, Gill David, Christie Patrick (2020). The COVID-19 pandemic, small-scale fisheries and coastal fishing communities. Coastal Management.

[CR6] Bianchetti Crtistina, Boano Camillo, Di Campli Antonio (2020). Thinking with quarantine urbanism?. Space and Culture.

[CR7] Booker, J. 2016. *Maritime quarantine: the British experience, c. 1650–1900*. Oxford: Routledge.

[CR8] Boyd, Matt, Baker, Michael. G., & Wilson, Nick. 2020. ‘Border closure for island nations? Analysis of pandemic and bioweapon‐related threats suggests some scenarios warrant drastic action.’ *Australian and New Zealand Journal of Public Health*, 44(2), 89. 10.1111/1753-6405.12991 PMC7262147 32259383

[CR9] Bremner Lindsay (2014). Folded ocean: The spatial transformation of the Indian ocean world. Journal of the Indian Ocean Region.

[CR10] Bremner Lindsay (2017). Observations on the concept of the aquapelago occasioned by researching the Maldives. Shima.

[CR11] Césaire, Aimé*.* 1969. *Une tempête [A Tempest],* Collection Théâtre*, 22*. Paris, France: Editions du Seuil.

[CR12] Christou, Prokopis A., & Farmaki, Anna. 2019. ‘Utopia as a reinforcement of tourist experiences.’ *Annals of Tourism Research*. DOI: 10.1016/j.annals.2018.11.003

[CR13] Conkling P (2007). On islanders and islandness. Geographical Review.

[CR14] De Alwis, Malathi. (2004). The moral mother syndrome. *Indian Journal of Gender Studies, 11*(1), 65-73.

[CR15] De Beukelaer, Christiaan. 2021. COVID-19 at sea: ‘the world as you know it no longer exists’. *Cultural Studies* 35, no. 2-3: 572-584.

[CR16] DeVotta, Neil. 2021.’Sri Lanka: the return to ethnocracy.’ *Journal of Democracy* 32(1): 96–110.

[CR17] Dure, Olivia. 2017. "Divine embodiment and women’s resistance in Sri Lanka: opposing the ideologies of nation and empire" (2017). Claremont Colleges Library Undergraduate Research Award. 6. http://scholarship.claremont.edu/cclura_2017/6. Accessed 22 Aug 2021.

[CR18] Engelmann, Lukas & Lynteris, Christos. 2020. *Sulphuric Utopias: a history of maritime fumigation*. Mass, MIT Press.

[CR19] Erikson Thomas H (1993). In which sense do cultural islands exist?. Social Anthropology.

[CR20] Fisher Mark (2012). What is hauntology?. Film Quarterly.

[CR21] Foucault, Michel. ‘Right of death and power over life.’ *The History of Sexuality: Volume 1*. Translated by Robert Hurley. London: Penguin Books Ltd., 1998.

[CR22] Gibson-Fall Fawzia (2021). Military responses to COVID-19, emerging trends in global civil-military engagements. Review of International Studies.

[CR23] Gillis, John. 2004. *Islands of the mind: how the human imagination created the Atlantic world*. London: Palgrave Macmillan.

[CR24] Godamunne, Vichitra. 2000. ‘Pandemic orientalism and some realities.’ *Medium*. Retrieved from https://medium.com/@vichitra.ksg/pandemic-orientalism-and-some-realities-76eca70455b6 (accessed 10/09/20).

[CR25] Grewal Kiran (2021). Privilege, precarity and the epistemic and political challenge of COVID-19. PORTAL Journal of Multidisciplinary International Studies.

[CR26] Grydehøj, Adam, Ilan Kelman, and Ping Su. 2020. Island geographies of separation and cohesion: The coronavirus (COVID‐19) pandemic and the geopolitics of Kalaallit Nunaat (Greenland). *Tijdschrift voor Economische en Sociale Geografie* 111 (3): 288–301. 10.1111/tesg.12423 PMC7323316 32836492

[CR27] Hau’ofa, Epeli. 2008. *We are the ocean: selected works*, 27- 40. Hawai’i: University of Hawai’i Press.

[CR28] Havice Elizabeth, Marschke Melissa, Vandergeest Peter (2020). Industrial seafood systems in the immobilizing COVID-19 moment. Agriculture and Human Values.

[CR29] Hayward, Philip. 2016. ‘Introduction: towards an expanded concept of island studies,’ *Shima: International Journal of Research into Island Cultures.*10.21463/shima.10.1.03.

[CR30] Herman, Doug. 2020. ‘Shutting down Hawai'i: a historical perspective on epidemics in the islands.’ Smithsonian Magazine, March 25, 2020. Available online: https://www.smithsonianmag.com/history/shutting-down-hawaii-historical-perspective-epidemics-islands-180974506/ (accessed 10.09.20).

[CR31] Howell Alison (2014). The global politics of medicine: Beyond global health, against securitisation theory. Review of International Studies.

[CR32] ICUA. 2015. ‘Island cities and urban achipelagoes’. Available online: http://www.islandcities.org/ (accessed 14/09/20).

[CR33] Ingram, Alan. 2009. The Geopolitics of Disease. *Geography Compass* 3: 2084–2097.

[CR34] Jazeel Tariq (2009). Reading the geography of Sri Lankan island-ness: Colonial repetitions, postcolonial possibilities. Contemporary South Asia.

[CR35] Kadirgamar Ahilan (2013). The question of militarisation in post-war Sri Lanka. Economic and Political Weekly.

[CR36] Kahrl, Andrew W. 2016 *The land was ours: how black beaches became white wealth in the coastal south*. UNC Press Books

[CR37] Kelly Catherine (2008). " Not from the college, but through the public and the legislature" Charles Maclean and the Relocation of Medical Debate in the Early Nineteenth Century. Bulletin of the History of Medicine.

[CR38] Klooster, Wim. 1998. *Illicit riches: Dutch trade in the Caribbean, 1648-1795*. New York: Brill.

[CR39] Lincoln Martha, Lincoln Bruce (2015). Toward a critical hauntology: Bare afterlife and the ghosts of Ba Chúc. Comparative Studies in Society and History.

[CR40] Luo Bin, Grydehøj Adam (2017). Sacred islands and island symbolism in Ancient and Imperial China: An exercise in decolonial island studies. Island Studies Journal.

[CR41] Meegama, Saman A. 2012. *Famine, fevers and fear: the state and disease in British Colonial Sri Lanka*. Colombo: Sridevi Publication.

[CR42] Mountz Alison (2011). ‘The enforcement archipelago: Detention, haunting, and asylum on islands. Political Geography.

[CR43] Murray, Melanie A. 2009. *Island paradise: the myth: an examination of contemporary Caribbean and Sri Lankan writing*. Rodopi: Brill.

[CR44] Osborne, H. 2020. ‘Scientists worked out the best island nations to survive a global pandemic in a study last year’, March, 12 2020, Newsweek, URL: https://www.newsweek.com/coronavirus-global-pandemic-island-nations-survive-1491897 (accessed 20/07/20).

[CR45] Payne, Christopher. 2014. *North brother island: the last unknown place in New York City*, New York: Fordham University Press.

[CR46] Pieris, Pradeep. 2021a. ‘Introduction: reflections on pandemic governance in Sri Lanka.’ In Peiris, P. (ed.), *Is the Cure Worse than the Disease: Reflections on COVID Governance in Sri Lanka*, Colombo Centre for Policy Alternatives, 11–27.

[CR47] Pieris, Pradeep. 2021b. ‘Healing the population by constructing subjects: pandemic governmentality of Sri Lanka’. In Peiris, P. (ed.), *Is the Cure Worse than the Disease: Reflections on COVID Governance in Sri Lanka*, Colombo Centre for Policy Alternatives, 61–86.

[CR48] *Quarantine and the prevention of diseases ordinance of Ceylon*. 1897. https://www.quarantine.health.gov.lk/images/pdf/act/quarantine_and_prevention_of_disease_act553.pdf (accessed 09/09/20).

[CR49] Sheller, Mimi. 2020. ‘Reconstructing tourism in the Caribbean: connecting pandemic recovery, climate resilience and sustainable tourism through mobility justice.’ *Journal of Sustainable Tourism*, 1-14. 10.1080/09669582.2020.1791141.

[CR50] Sikka, Tina. 2020. Racialisation, COVID-19 and bioessentialism, Transforming Society, May 29, 2020. available online: http://www.transformingsociety.co.uk/2020/05/29/racialisation-covid-19-and-bioessentialism/. Accessed 18 Sep 2020.

[CR51] Siriwardane-de Zoysa, Rapti. 2021b. ‘Decolonizing seascapes: imaginaries and absences on an island hub.’ *Postcolonial Interventions* VI(I). 10.5281/zenodo.4483976

[CR52] Siriwardane-de Zoysa, Rapti; Sondang, Irene. F. and Ganda Purnama, Arif. 2021a. ‘Opto-haptic fieldwork encounters in pandemic Southeast Asia.’ Society for Cultural Anthropology – *Fieldsights/ Members Voices.* Available online: https://culanth.org/fieldsights/opto-haptic-fieldwork-encounters-in-pandemic-southeast-asia (accessed on 02.10.2021).

[CR53] Sivasundaram, Sujit. 2013. *Islanded: Britain, Sri Lanka and the Bounds of an Indian Ocean Colony*, Chicago & London: University of Chicago Press.

[CR54] Sridhar, Aarthi and Jalais, Annu. 2021. 2021. ‘A collaboratory for Indian ocean ethnographies.’ Society for Cultural Anthropology – *Fieldsights/ Members Voices.* Available online: https://culanth.org/fieldsights/series/a-collaboratory-of-indian-ocean-ethnographies (accessed on 02.10.2021).

[CR55] Suwa, Jun'ichiro. 2007. ‘The space of Shima.’ *Shima: The International Journal of Research into Island Cultures*, *1*(1), 6–14.

[CR56] Tan, Kevin S.Y.; Lim, Grace. 2021. *Populations, precarity and pandemics: the demographics of inequality and COVID-19 in Southeast Asia*. ISEAS Yusof Ishak Institute. Available online: https://think-asia.org/handle/11540/13253 (accessed on 05.01.2022).

[CR57] Tuncbilek Gonca Z (2020). Quarantine(d) space: Urla-Izmir (Smyrna) Island. Space and Culture.

[CR58] Wickramasinghe Nira (2010). In Sri Lanka, the triumph of vulgar patriotism. Current History.

